# Supplementary material for: 12q21 Interstitial Deletions: Seven New Syndromic Cases Detected by Array-CGH and Review of the Literature
Source: Genes (Basel). 2022 Apr 27;13(5):780. doi: 10.3390/genes13050780 (PMC9141874; doi:10.3390/genes13050780)
Supplement: Supplementary file 1 [file genes-13-00780-s001.zip › genes-1654880-supplementary.pdf]

Supplementary Table S1: List of the most relevant coding genes in 12q21 region.

**HUMAN GENE SYMBOL**	**GENE FULL NAME**	**MAIN BIOLOGICAL ACTIVITY**	**OMIM DISEASE ASSOCIATION**	**HI⩽** 10% [13]	**pLI ⩾ 0.9** [13,15]
ZFC3H1	Zinc finger, C3H1-type containing	Unknown			X
TPH2	Tryptophan hydroxylase 2	Biosynthesis of serotonin	Susceptibility to: MAJOR DEPRESSIVE DISORDER (MDD) [608516];ATTENTION DEFICIT–HYPERACTIVITY DISORDER type 7 (ADHD7) [613003]		
TRHDE	Thyrotropin-releasing hormone degrading enzyme	Cleaves and inactivates the neuropeptide thyrotropin-releasing hormone		X	
KRR1	Small subunit (SSU) processome component	40S ribosome biogenesis		X	
NAP1L1	Nucleosome assembly protein 1-like 1	May be involved in modulating chromatin formation and cell proliferation		X	X
BBS10	Bardet-Biedl syndrome 10	Assists the folding of proteins upon ATP hydrolysis	BARDET-BIEDL SYNDROME 10 [615987]		
ZDHHC17	Zinc finger, DHHC-type containing 17	Palmitoyltransferase specific for a subset of neuronal proteins, including SYT1		X	X
E2F7	E2F transcription factor 7	Regulates cell cycle progression			X
NAV3	Neuron navigator 3	Belongs to neuron navigator family and is expressed predominantly in the nervous system			X
**SYT1**	Synaptotagmin I	Interacts with membrane during trafficking of synaptic vesicles at the active zone of the synapse	BAKER-GORDON SYNDROME [618218]	X	
**PPP1R12A**	Protein phosphatase 1, regulatory subunit 12A	Key regulator of protein phosphatase 1C (PPP1C). Mediates binding to myosin.	GENITOURINARY AND/OR BRAIN MALFORMATION SYNDROME [618820]	X	X
**OTOGL**	Otogelin-like	Expressed in the inner ear of vertebrates	DEAFNESS 84B [614944]		
PTPRQ	Protein tyrosine phosphatase, receptor type, Q	Phosphatidylinositol phosphatase required for auditory function	DEAFNESS 73 [617663]; DEAFNESS 84 [613391]		
MYF6	Myogenic factor 6 (herculin)	Probable basic helix-loop-helix DNA binding protein involved in muscle differentiation		X	

MYF5	Myogenic factor 5	Muscle differentiation	OPHTHALMOPLEGIA, EXTERNAL, WITH RIB AND VERTEBRAL ANOMALIES [618155]	X	
PPFIA2	Protein tyrosine phosphatase, receptor type, F polypeptide (PTPRF) interacting protein	Regulator of higher-order brain functions in mammals			X
**TMTC2**	Transmembrane and tetratricopeptide repeat containing 2	Unknown		X	
**ALX1**	ALX homeobox 1	Unknown	FRONTONASAL DYSPLASIA 3 [613456]	X	
**NTS**	Neurotensin	Endocrine/paracrine role in the regulation of fat metabolism.		X	
**CEP290**	Centrosomal protein 290kDa	Localization of ciliary and photo-transduction proteins in retinal photoreceptor cells	JOUBERT syndrome type 5 (JBTS5) [610188]; SENIOR-LOKEN syndrome type 6 (SLSN6) [610189]; MECKEL SYNDROME type 4 (MKS4) [611134]		
**TMTC3**	Transmembrane and tetratricopeptide repeat containing 3	Unknown	LISSENCEPHALY 8 [617255]		
**KITLG**	KIT ligand	Regulation of cell survival and proliferation	DEAFNESS 69 UNILATERAL OR ASYMMETRIC [616697]	X	
DUSP6	Dual specificity phosphatase 6	Negative regulator of members of the mitogen-activated protein (MAP) kinase superfamily	HYPOGONADOTROPIC HYPOGONADISM 19 WITH OR WITHOUT ANOSMIA [615269]	X	X
ATP2B1	ATPase, Ca++ transporting, plasma membrane 1	Intracellular calcium homeostasis		X	X
**KERA**	Keratocan	Keratan sulfate proteoglycan involved in corneal transparency	CORNEA PLANA 2 [217300]		
**DCN**	Decorin	Collagen fibril assembly	CORNEAL DYSTROPHY [610048]	X	

Gene symbol and full name, main known biological activity [12], OMIM disease association [14], if applicable, HI score ⩽ 10% and/or pLI score ⩾ **0.9** [13,15] (see the main text for the listed criteria). Genes mentioned in the main text are in bold font.

12. UCSC Genome Browser. Available online: http://genome-euro.ucsc.edu/index.html (accessed on 15 January 2022).
13. DECIPHER. Available on line: https://www.deciphergenomics.org/ (accessed on 15 January 2022).
14. OMIM. Available online: https://www.omim.org/ (accessed on 15 January 2022).
15. gnomAD. Available online: https://gnomad.broadinstitute.org/ (accessed on 15 January 2022).
